# Supplementary material for: L-Arginine prevents cereblon-mediated ubiquitination of glucokinase and stimulates glucose-6-phosphate production in pancreatic β-cells
Source: Commun Biol. 2020 Sep 8;3:497. doi: 10.1038/s42003-020-01226-3 (PMC7479149; doi:10.1038/s42003-020-01226-3)
Supplement: Supplementary file 1 — Supplemental Information [file 42003_2020_1226_MOESM1_ESM.docx]

**Supplementary Information for**

***L*-Arginine prevents glucokinase from cereblon-mediated ubiquitination of glucokinase and stimulates glucose-6-phosphate production in pancreatic β-cells**

**This pdf file includes:**

Abbreviation

Supplemental Figure 1, 2, 3, 4, and 5.

The Supplemental Data Set 1 includes

Original photo of Figure 1d, 1e, 2e, 3e, 5a, 5b and 5c

The Supplemental Data Set 2 includes

Original OD values of Figure 2a, 5h, Supplemental Figure 1b and 5e

**Abbreviation**

ADP Adenosine diphosphate

ATP Adenosine triphosphate

CAT Cationic amino acid transporter

CRBN Cereblon

DMF Dimethylformamide

EDGE Ethylene glycol diglycidyl ether

ER Endoplasmic reticulum

G6P Glucose-6-phosphate

GCK Glucokinase

GKRP Glucokinase regulatory protein

GSIS Glucose stimulated insulin secretion

HA Hemagglutinin

HXK1 Hexokinase 1

HOMAβ Homeostatic model assessment of β cell

IMiDs Immunomodulatory imide drugs

MODY Maturity-onset diabetes of the young

MS Mass spectrometry

OD Optical density

PFK Phosphofructokinase

PROTACs Proteolysis targeting chimeras

SLC7A1-2 Solute carrier family 7 member 1-2

UIM Ubiquitin interacting motif

UGGT1 UDP-glucose:glycoprotein glucosyltransferase 1.

WT Wild-type


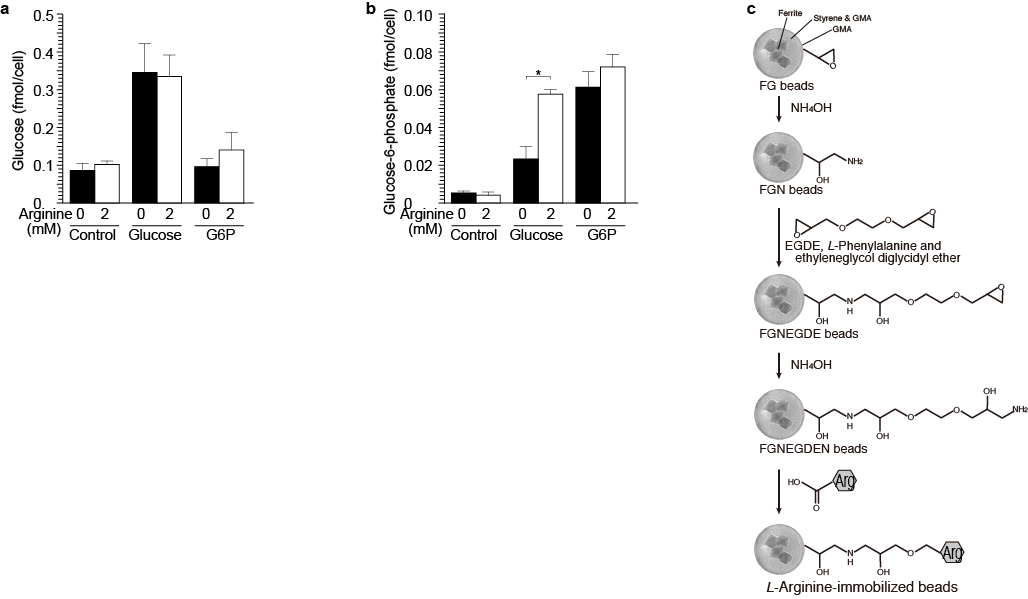


**Supplemental Figure 1. Arginine binds to glucokinase. (Supportive data for Fig. 1)**

**a** and **b**, Intracellular glucose and glucose-6-phosphate (G6P) contents were determined by mass spectrometry. *L*-Arginine increased intracellular G6P production from glucose and exogenous G6P increased intracellular G6P (supportive data for Fig. 1a, b).

**c**, Preparation of *L*-arginine-immobilized magnetic nanobeads. Epoxy groups on FG beads were aminolyzed by NH_4_OH (FGN beads) and coupled to ethylene glycol diglycidyl ether (EGDE) to produce FGNEGDE beads. Epoxy groups on FGNEGDEN beads were aminolyzed by NH_4_OH to produce FGNEGDEN beads. FGNEGDEN beads were then coupled with carboxyl groups of *L*-arginine in dimethylformamide (DMF) containing EDC, trimethylamine, and DMAP (supportive data for Fig. 1c).


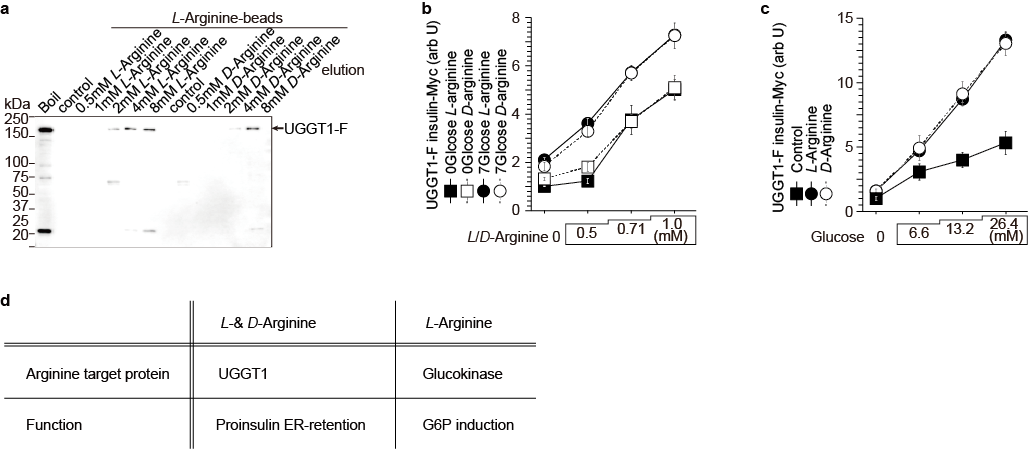


**Supplemental Figure 2. *L*-Arginine stimulates glucose-6-phosphate and insulin production. (Supportive data for Fig. 2)**

**a**, UGGT1 binds to both *L*- and *D*-arginine *in vitro*. In contrast, glucokinase binds to only *L*-arginine (Fig. 2e). Data is from Fig. 2b of Cho *et. al.*^26^.

**b** and **c**, *L*- and *D*-arginine simulate UGGT1 mediated insulin secretion similarly. Data represent the mean ± S.E. (n=3-5).

**d**, Two pathways by which arginine induces insulin secretion. *L*-/*D*-Arginine binds to UGGT1 in the ER^26^ and release proinsulin from retention at the ER. *L*-Arginine binds to glucokinase and stimulates G6P production that leads to insulin secretion.


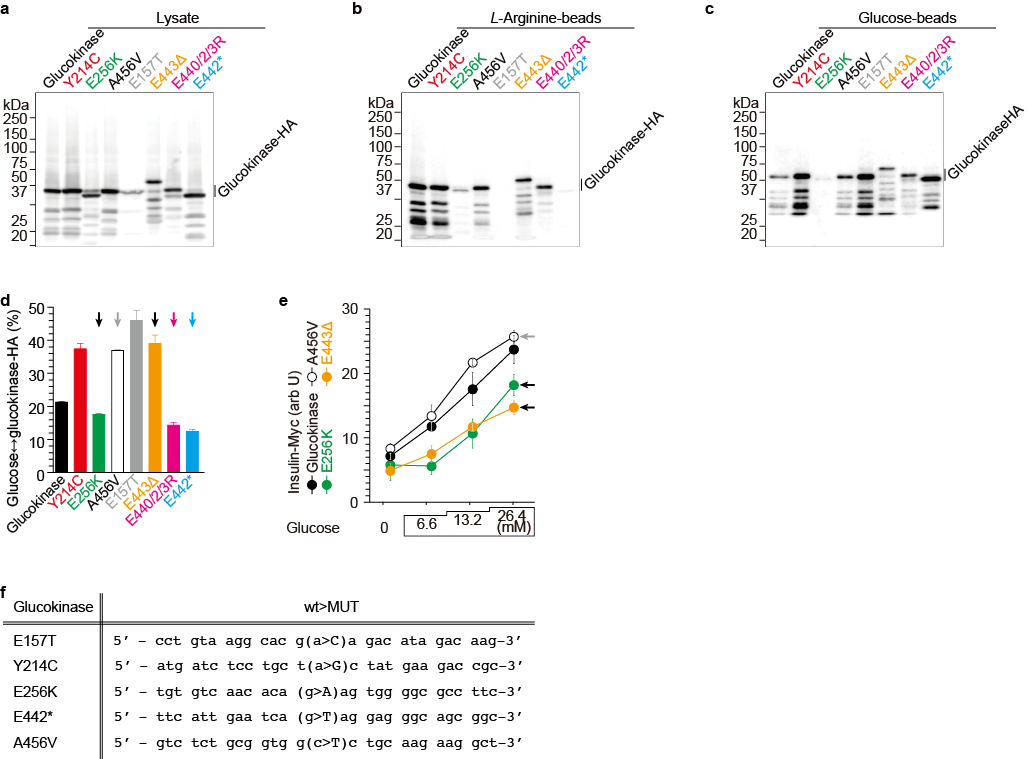


**Supplemental Figure 3. E256, E442, and E443 residues are involved in arginine-induced insulin secretion in NIT-1 β-cells. (Supportive data for Fig. 3)**

**a**-**c**, Lysate (**a**), *L*-arginine binding fraction (**b**), and glucose-binding fractions (**c**) of cells expressing WT and mutant glucokinase.

**d**, Reduced binding of glucose to E256K, E440/442K, E440/2/3R, and E442* glucokinase mutants when several glucokinase proteins were analyzed using glucose-immobilized beads (Supplemental Fig. 2c).

**e**, Impaired glucose-induced insulin secretion in NIT-1 cells expressing E256K and E443Δ mutant glucokinase. Data represent the mean ± S.E. (n=3-5).

**f**, Sequence of glucokinase mutants.


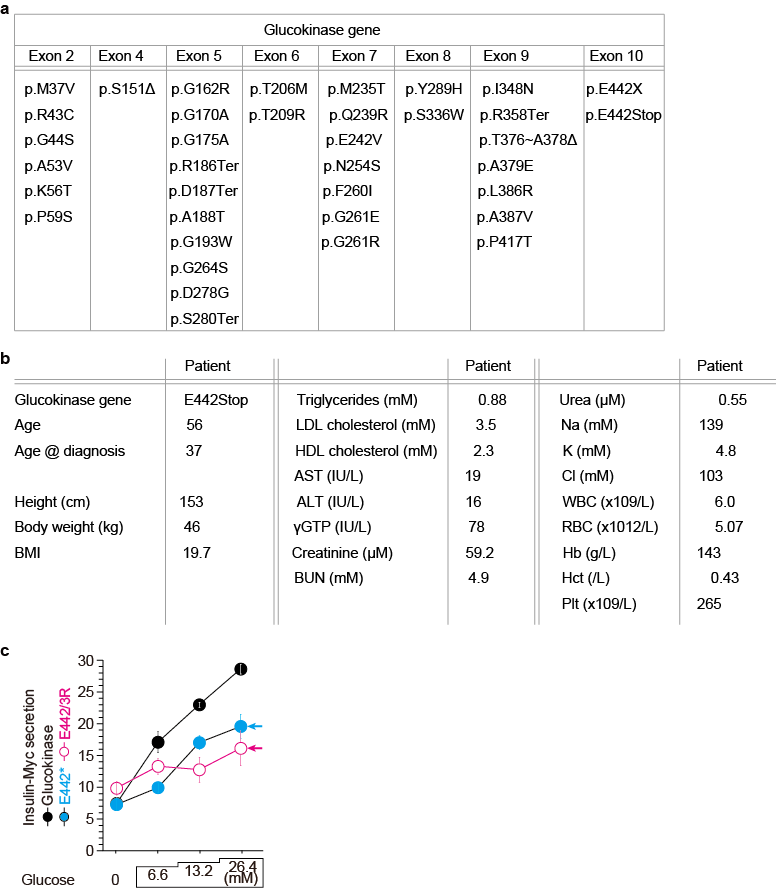


**Supplemental Figure 4. Impaired insulin secretion in a subject with glucokinase^E442*^. (Supportive data for Fig. 4)**

**a**, List of MODY2 mutations^27-29^.

**b**, Clinical parameters of the MODY2 (GCK^E442*^) patient are listed.

**c**, Impaired glucose-induced insulin secretion in NIT-1 cells transfected with pCDNA-GCK-HA with E442* and E442/3R mutations along with insulin expression vector. Data represent the mean ± S.E. (n=3-5).

**
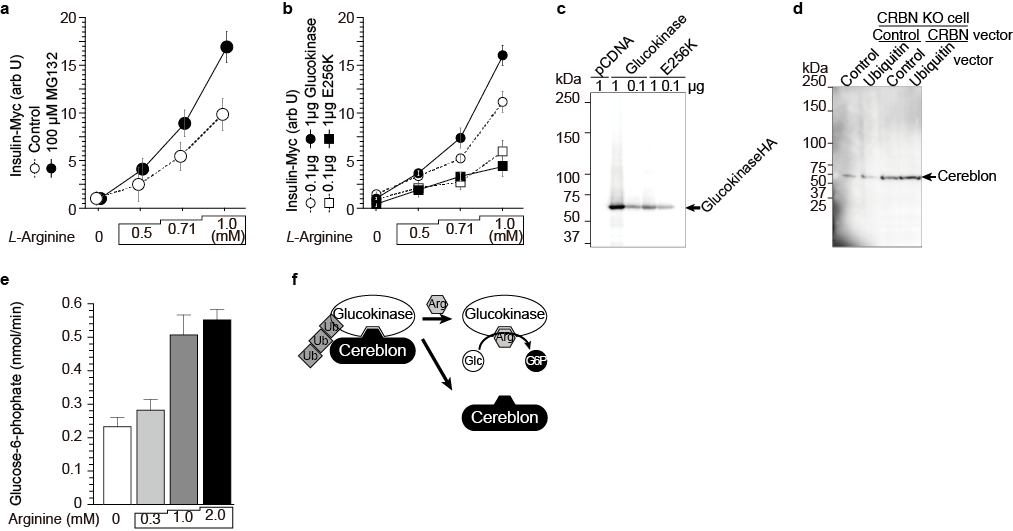
**

**Supplemental Figure 5. *L*-Arginine protects glucokinase from ubiquitination and stimulates insulin secretion. (Supportive data for Fig. 5)**

**a**, Arginine increases insulin secretion more when proteasomal degradation of glucokinase is prevented. Arginine-induced insulin secretion was measured with/without a protease inhibitor MG132. Data represent the mean ± S.E. (n=3-5).

**b** and **c**, Overexpression of WT glucokinase dose dependently increases arginine-induced insulin secretion. Arginine induced insulin secretion was measured in NIT-1 cells expressing low and high amounts of glucokinase expression vectors. E256K mutated glucokinase induced insulin secretion less. Glucokinase and E256 protein expression was confirmed by WB (**c**). Data represent the mean ± S.E. (n=3-5).

**d**, Western blot of cereblon in control and cereblon KO HEK293 cells. Supporting data for Fig. 5c.

**e**, Arginine stimulates activity of recombinant glucokinase protein *in vitro*. (n=6). HEK293FT cells that show high transfection rate and express SV40 large T antigen were transfected with glucokinase expression vector pSG5-glucokinase with SV40 replication origin and the transfected cells were harvested after 36 h. Arginine was administered to the lysate and glucose-6-phosphate production was analyzed.

**f**, Model of *L*-arginine-induced insulin secretion. In the absence of arginine, glucokinase binds to cereblon promoting ubiquitination by cereblon. Arginine binds to glucokinase and competes with cereblon to prevent ubiquitination. Binding of arginine to glucokinase phosphorylates glucose and stimulates insulin secretion.
