# Supplementary material for: L-Arginine prevents cereblon-mediated ubiquitination of glucokinase and stimulates glucose-6-phosphate production in pancreatic β-cells
Source: Commun Biol. 2020 Sep 8;3:497. doi: 10.1038/s42003-020-01226-3 (PMC7479149; doi:10.1038/s42003-020-01226-3)
Supplement: Supplementary file 2 — Description of Additional Supplementary Files [file 42003_2020_1226_MOESM2_ESM.pdf]

Description of Additional Supplementary Files

Supplementary Data 1: includes original photo of Figure 1d, 1e, 2e, 3e, 5a, 5b and 5c

Supplementary Data 2: includes original OD values of Figure 2a, 5h, Supplemental Figure 1b and 5e.
