# Supplementary figures and images for: L-Arginine prevents cereblon-mediated ubiquitination of glucokinase and stimulates glucose-6-phosphate production in pancreatic β-cells
Source: Commun Biol. 2020 Sep 8;3:497. doi: 10.1038/s42003-020-01226-3 (PMC7479149; doi:10.1038/s42003-020-01226-3)

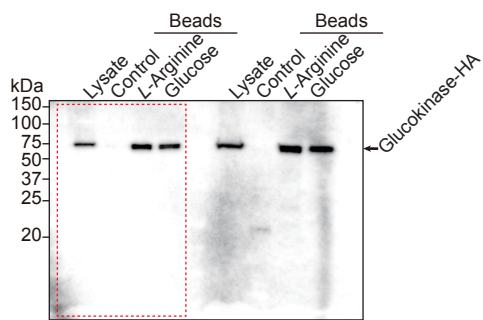

Fig. 1d

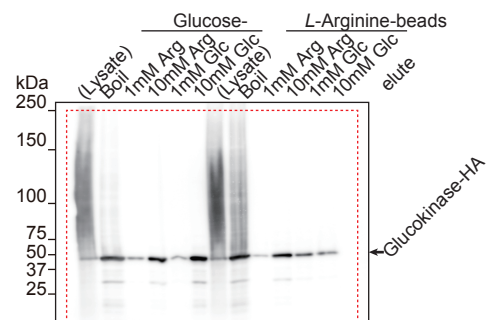

Fig. 1e

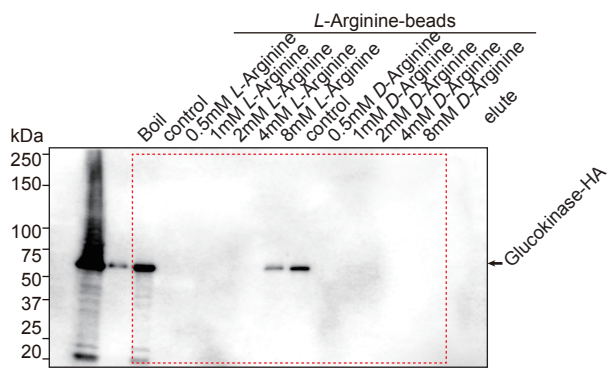

Fig. 2e

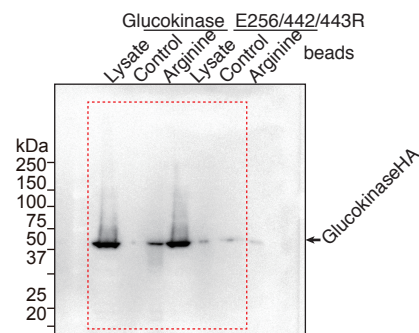

Fig. 3e

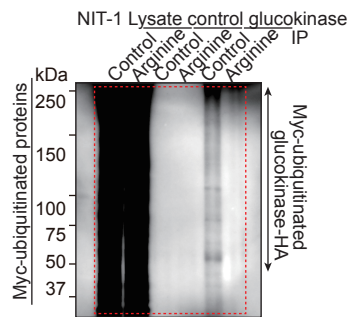

Fig. 5a

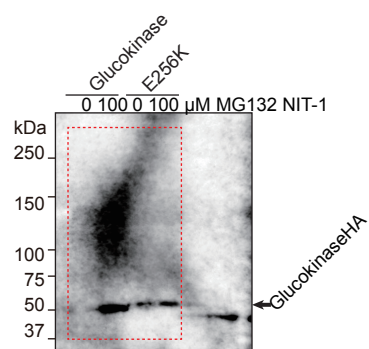

Fig. 5b

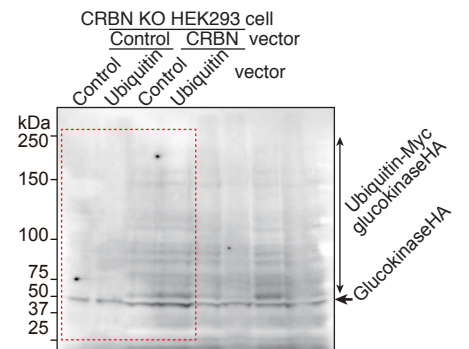

Fig. 5c

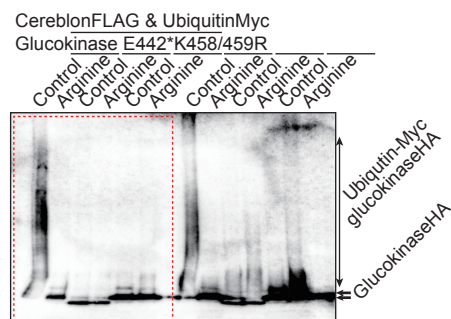

Fig. 5d

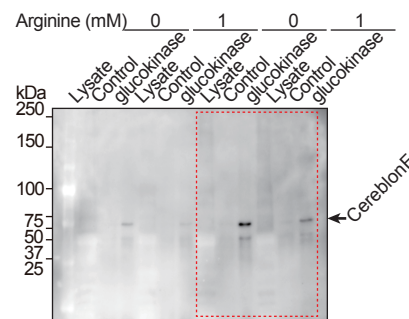

Fig. 5g

Supplement: Supplementary file 3 — Supplementary Data 1 [file 42003_2020_1226_MOESM3_ESM.pdf]
