# Supplementary material for: L-Arginine prevents cereblon-mediated ubiquitination of glucokinase and stimulates glucose-6-phosphate production in pancreatic β-cells
Source: Commun Biol. 2020 Sep 8;3:497. doi: 10.1038/s42003-020-01226-3 (PMC7479149; doi:10.1038/s42003-020-01226-3)
Supplement: Supplementary file 5 — Reporting Summary [file 42003_2020_1226_MOESM5_ESM.pdf]

nature research

Corresponding author(s): Takeshi Imai

Last updated by author(s): Jul 15, 2020

Reporting Summary

Nature Research wishes to improve the reproducibility of the work that we publish. This form provides structure for consistency and transparency in reporting. For further information on Nature Research policies, see our Editorial Policies and the Editorial Policy Checklist.

Statistics

For all statistical analyses, confirm that the following items are present in the figure legend, table legend, main text, or Methods section.

n/a	Confirmed	
☐	☒	The exact sample size (*n*) for each experimental group/condition, given as a discrete number and unit of measurement
☐	☒	A statement on whether measurements were taken from distinct samples or whether the same sample was measured repeatedly
☐	☒	The statistical test(s) used AND whether they are one- or two-sided<br>*Only common tests should be described solely by name; describe more complex techniques in the Methods section.*
☐	☒	A description of all covariates tested
☒	☐	A description of any assumptions or corrections, such as tests of normality and adjustment for multiple comparisons
☐	☒	A full description of the statistical parameters including central tendency (e.g. means) or other basic estimates (e.g. regression coefficient) AND variation (e.g. standard deviation) or associated estimates of uncertainty (e.g. confidence intervals)
☐	☒	For null hypothesis testing, the test statistic (e.g. *F*, *t*, *r*) with confidence intervals, effect sizes, degrees of freedom and *P* value noted<br>*Give P values as exact values whenever suitable.*
☒	☐	For Bayesian analysis, information on the choice of priors and Markov chain Monte Carlo settings
☒	☐	For hierarchical and complex designs, identification of the appropriate level for tests and full reporting of outcomes
☒	☐	Estimates of effect sizes (e.g. Cohen's *d*, Pearson's *r*), indicating how they were calculated

Our web collection on statistics for biologists contains articles on many of the points above.

Software and code

Policy information about availability of computer code

Data collection	Immune-complexes were detected using can get signal (TOYOBO) and Immunostar LD (Wako) substrate. Signals were quantified with the LAS 4000 imaging system (GE healthcare). ImageJ was used for densitometry of scanned membrane. Data of secreted insulin was detected ELISA was calculated with Excel and STATVIEW.
Data analysis	Data calculation is using STATVIEW and Excel.

For manuscripts utilizing custom algorithms or software that are central to the research but not yet described in published literature, software must be made available to editors and reviewers. We strongly encourage code deposition in a community repository (e.g. GitHub). See the Nature Research guidelines for submitting code & software for further information.

Data

Policy information about availability of data

All manuscripts must include a data availability statement. This statement should provide the following information, where applicable:

- Accession codes, unique identifiers, or web links for publicly available datasets
- A list of figures that have associated raw data
- A description of any restrictions on data availability

There is no restriction on data availability.

Field-specific reporting

Please select the one below that is the best fit for your research. If you are not sure, read the appropriate sections before making your selection.

☒ Life sciences ☐ Behavioural & social sciences ☐ Ecological, evolutionary & environmental sciences

For a reference copy of the document with all sections, see nature.com/documents/nr-reporting-summary-flat.pdf

Life sciences study design

All studies must disclose on these points even when the disclosure is negative.

Sample size	Analysis in cells ana in vitro;<br>No statistical analysis; n=3~7<br>Statistical analysis; n= 6~
Data exclusions	No data were excluded from the analysis which is on the work.
Replication	For reproducibility of key experiments, we performed total number of experiments exceed 10 times including experiments for setting conditions with similar results. Also, we employed multiple approaches to confirm one result.
Randomization	Randomization and blinding analysis in GCK mutated analysis.<br>Random sampling in the MODY patient study.
Blinding	Randomization and blinding analysis in GCK mutated analysis.<br>Bliding analysis in the MODY patient study.

Reporting for specific materials, systems and methods

We require information from authors about some types of materials, experimental systems and methods used in many studies. Here, indicate whether each material, system or method listed is relevant to your study. If you are not sure if a list item applies to your research, read the appropriate section before selecting a response.

Materials & experimental systems

n/a	Involved in the study	
☐	☒	Antibodies
☐	☒	Eukaryotic cell lines
☒	☐	Palaeontology and archaeology
☒	☐	Animals and other organisms
☐	☒	Human research participants
☐	☒	Clinical data
☐	☐	Dual use research of concern

Methods

n/a	Involved in the study	
☒	☐	ChIP-seq
☒	☐	Flow cytometry
☒	☐	MRI-based neuroimaging

Antibodies

Antibodies used	The following antibodies were purchased: insulin (L6B10, Cell Signaling Technology, Danvers, MA, USA and sc-9168, Santa Cruz, California CA, USA), GM130 (cis-Golgi network staining, 610822, BD Transduction Laboratories, New York, NY, USA), FLAG (F-1804, Sigma, St Louis, MO, USA), HA (anti-HA high affinity 3F10, Roche, Basel, Switzerland), KDEL (ER staining, SPA-827, Stressgen, Victoria, BC, Canada), and Alexa Fluor-conjugated secondary antibodies (Thermo Fisher, Rockford, IL, USA).
Validation	Insulin (L6B10, Cell Signaling Technology, Danvers, MA, USA and sc-9168, Santa Cruz, California CA, USA),>>ELISA, IP, WB, ICC<br>GM130 (cis-Golgi network staining, 610822, BD Transduction Laboratories, New York, NY, USA), > ICC<br>FLAG (F-1804, Sigma, St Louis, MO, USA), > IP, WB<br>HA (anti-HA high affinity 3F10, Roche, Basel, Switzerland), >IP, WB<br>KDEL (ER staining, SPA-827, Stressgen, Victoria, BC, Canada),>IP, WB<br>Alexa Fluor-conjugated secondary antibodies (Thermo Fisher, Rockford, IL, USA). > ICC, WB

Eukaryotic cell lines

Policy information about cell lines

Cell line source(s)	Mouse pancreas-derived NIT1 cells<br>Human embryo kidney (HEK293 cells)

Authentication	NIT-1> CRL-2055 ATCC<br>HEK293 cells from Invitrogen
Mycoplasma contamination	NIT-1 was tested ATCC<br>HEK293 cells was tested Invitrogen
Commonly misidentified lines<br>(See ICLAC register)	HEK misidentified by HeLa by ICLAC ver 8.

Human research participants

Policy information about studies involving human research participants

Population characteristics	We examined GCK sequence of 489 subjects from 155 medical institutions who was suspected to have MODY (male/female, 266/223; age of onset, 16.3 ± 8.1 years; body mass index [BMI], 22.9 ± 5.3 kg/m2)
Recruitment	They are patients.
Ethics oversight	Arginine tolerance test in two MODY2 subjects was performed. Blood samples were obtained via a catheter inserted into a superficial forearm vein. An equivalent of 5 g arginine as 10% arginine hydrochloride (5 g) was administered as an intravenously over 30 s, the end of which was designated as time point 0. Blood samples were withdrawn at 3 and 5 min for the determination of insulin levels.<br>The clinical study of arginine tolerance test protocol was approved by the Institutional Review Board. Written informed consent was obtained from all participants.

Note that full information on the approval of the study protocol must also be provided in the manuscript.

Clinical data

Policy information about clinical studies

All manuscripts should comply with the ICMJE guidelines for publication of clinical research and a completed CONSORT checklist must be included with all submissions.

Clinical trial registration	The clinical study of arginine tolerance test protocol was approved by the Institutional Review Board of University (No. 25-153). Written informed consent was obtained from all participants.
Study protocol	Arginine tolerance test in two MODY2 subjects was performed at Hospital. Blood samples were obtained via a catheter inserted into a superficial forearm vein. An equivalent of 5 g arginine as 10% arginine hydrochloride (5 g) was administered as an intravenously over 30 s, the end of which was designated as time point 0. Blood samples were withdrawn at 3 and 5 min for the determination of insulin levels.
Data collection	An equivalent of 5 g arginine as 10% arginine hydrochloride (5 g) was administered as an intravenously over 30 s, the end of which was designated as time point 0. Blood samples were withdrawn at 3 and 5 min for the determination of insulin levels.
Outcomes	Circulating concentration of glucose, insulin, C-peptide.

Dual use research of concern

Policy information about dual use research of concern

Hazards

Could the accidental, deliberate or reckless misuse of agents or technologies generated in the work, or the application of information presented in the manuscript, pose a threat to:

No	Yes	
☒	☐	Public health
☒	☐	National security
☒	☐	Crops and/or livestock
☒	☐	Ecosystems
☒	☐	Any other significant area

Experiments of concern

Does the work involve any of these experiments of concern:

No	Yes	
☒	☐	Demonstrate how to render a vaccine ineffective
☒	☐	Confer resistance to therapeutically useful antibiotics or antiviral agents
☒	☐	Enhance the virulence of a pathogen or render a nonpathogen virulent
☒	☐	Increase transmissibility of a pathogen
☒	☐	Alter the host range of a pathogen
☒	☐	Enable evasion of diagnostic/detection modalities
☒	☐	Enable the weaponization of a biological agent or toxin
☒	☐	Any other potentially harmful combination of experiments and agents
